# Insights into the Interaction Mechanism of DTP3 with MKK7 by Using STD-NMR and Computational Approaches

**DOI:** 10.3390/biomedicines9010020

**Published:** 2020-12-30

**Authors:** Annamaria Sandomenico, Lorenzo Di Rienzo, Luisa Calvanese, Emanuela Iaccarino, Gabriella D’Auria, Lucia Falcigno, Angela Chambery, Rosita Russo, Guido Franzoso, Laura Tornatore, Marco D’Abramo, Menotti Ruvo, Edoardo Milanetti, Domenico Raimondo

**Affiliations:** 1 Institute of Biostructures and Bioimaging (IBB)-CNR, Via Mezzocannone 16, 80134 Naples, Italy; annamaria.sandomenico@gmail.com (A.S.); emanuela.iaccarino@unicampania.it (E.I.); gabriella.dauria@unina.it (G.D.); falcigno@unina.it (L.F.); menotti.ruvo@unina.it (M.R.); 2 Center for Life Nano Science@Sapienza, Italian Institute of Technology, Viale Regina Elena 291, 00161 Rome, Italy; lorenzo.dirienzo@uniroma1.it; 3 CIRPeB, University of Naples Federico II, 80134 Naples, Italy; luisa.calvanese@unina.it; 4 Department of Pharmacy, University of Naples “Federico II”, Via Mezzocannone 16, 80134 Naples, Italy; 5 Department of Environmental, Biological and Pharmaceutical Sciences and Technologies, University of Campania Luigi Vanvitelli, 81100 Caserta, Italy; angela.chambery@unicampania.it (A.C.); rosita.russo@unicampania.it (R.R.);; 6 Centre for Molecular Immunology and Inflammation, Department of Immunology and Inflammation, Imperial College London, London W12 0NN, UK; g.franzoso@imperial.ac.uk (G.F.); la.tornatore@gmail.com (L.T.); 7 Department of Chemistry, Sapienza University of Rome, Piazzale Aldo Moro 5, 00185 Rome, Italy; marco.dabramo@uniroma1.it; 8 Department of Physics, Sapienza University of Rome, Piazzale Aldo Moro 5, 00185 Rome, Italy; 9 Department of Molecular Medicine, Sapienza University of Rome, Viale Regina Elena 291, 00161 Rome, Italy

**Keywords:** GADD45β, MKK7, multiple myeloma, protein-ligand interaction, STD-NMR

## Abstract

GADD45β/MKK7 complex is a non-redundant, cancer cell-restricted survival module downstream of the NF-kB survival pathway, and it has a pathogenically critical role in multiple myeloma, an incurable malignancy of plasma cells. The first-in-class GADD45β/MKK7 inhibitor DTP3 effectively kills MM cells expressing its molecular target, both in vitro and in vivo, by inducing MKK7/JNK-dependent apoptosis with no apparent toxicity to normal cells. DTP3 combines favorable drug-like properties, with on-target-specific pharmacology, resulting in a safe and cancer-selective therapeutic effect; however, its mode of action is only partially understood. In this work, we have investigated the molecular determinants underlying the MKK7 interaction with DTP3 by combining computational, NMR, and spectroscopic methods. Data gathered by fluorescence quenching and computational approaches consistently indicate that the N-terminal region of MKK7 is the optimal binding site explored by DTP3. These findings further the understanding of the selective mode of action of GADD45β/MKK7 inhibitors and inform potential mechanisms of drug resistance. Notably, upon validation of the safety and efficacy of DTP3 in human trials, our results could also facilitate the development of novel DTP3-like therapeutics with improved bioavailability or the capacity to bypass drug resistance.

## 1. Introduction

Mitogen-activated protein (MAP) kinases are evolutionarily conserved serine–threonine protein kinases that are activated in response to a wide variety of extracellular or intracellular stimuli. They play a key role in a number of intracellular networks where they transduce and integrate cellular signals into complex cytoplasmatic and nuclear processes, including proliferation, differentiation, and apoptosis [1,2]. These MAP kinases are activated by dual phosphorylation events on threonine and tyrosine residues within a Thr-Xaa-Tyr motif, which are systematically mediated by three-tiered cascades consisting of a MAP kinase (MAPK), a MAP kinase kinase (MAPKK, MEK, or MKK), and a MAP kinase kinase kinase (MAPKKK, MEKK, or MKKK). Ultimately, this signaling cascade results in the activation of the three major MAPKs pathways: the c-Jun N-terminal kinase (JNK) pathway, the p38 MAP kinase pathway, and the extracellular signal-regulated kinase (ERK) pathway [1,2]. Among MAPKKs, MKK7 is one of two the essential regulators of the JNK signaling and has been recently suggested to represent an emerging therapeutic target in cancer [3]. MKK7 consists of three domains: the D (docking) domain (residues 37–57), which contains a conserved docking site and is required for the binding to MAPK substrates; the kinase domain; and the DVD (domain for versatile docking) domain [2]. The MKK7 N-terminal D domain is involved in the binding to, and activation of, JNK. The central kinase domain (residues 120–380) contains a Ser–Xaa–Ala–Lys–Thr (S–X–A–K–T) kinase motif that is phosphorylated by upstream MKKKs. The C-terminal DVD domain, located at residues 377–400, plays an important role in the docking of upstream MAP3Ks such as MLKs, ASKs, TAKs, and LZK.

The activity of MKK7 is also regulated by direct physical interactions with several scaffold proteins (i.e., JIP proteins) and other partners (i.e., GADD45β and TIPRL), which play a crucial role in controlling the binding duration and signal intensity of MAP3K/MAP2K/MAPK complexes in the MAPK pathway [1,2,3,4]. We have recently identified the interaction between the NF-kB-regulated antiapoptotic factor, GADD45β, and the JNK kinase, MKK7, as a therapeutic target in multiple myeloma (MM), an incurable plasma cell malignancy accounting for about 2% of all cancer deaths and representing a paradigm for NF-kB-driven cancers [2,5]. GADD45β is upregulated in MM cells by NF-kB, associates with poor outcome in patients, and promotes myeloma cell survival by suppressing proapoptotic MKK7/JNK signaling [5].

**Figure 1:**
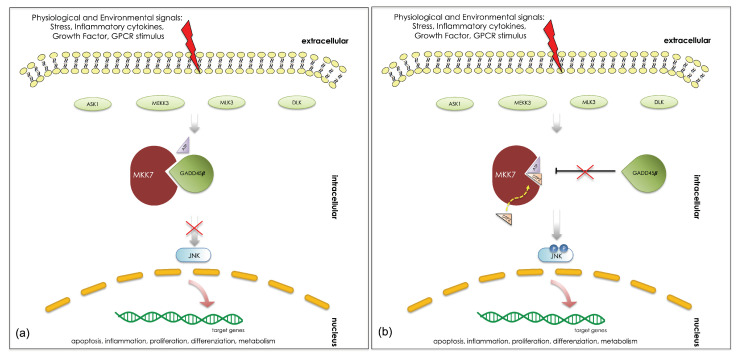
The GADD45β-regulated MKK7 signaling cascade in absence (**a**) and in presence (**b**) of the DTP3 peptide inhibitor. DTP3 interaction with MKK7 restores its kinase activity and the JNK-mediated apoptotic signal by preventing the GADD45β inhibition.

GADD45β mediates this activity by interacting directly with MKK7 and blocking its enzymatic activity by engaging the enzyme catalytic pocket, thereby preventing the access of ATP (See Figure 1a) [6,7]. DTP3 is a D-tripeptide of sequence Ac-D-Tyr-D-Arg-D-Phe-NH2 that has been identified through the screening of a combinatorial library of synthetic peptides targeting the GADD45β/MKK7 complex (See Figure 1b) [5,8,9,10]. By undertaking a drug discovery approach, we developed DTP3, which disrupts the GADD45β/MKK7 interaction and kills MM cells effectively by inducing MKK7/JNK-dependent apoptosis, without overt toxicity to normal tissues [5]. Preclinical studies demonstrated that the first-in-class GADD45β/MKK7 inhibitor DTP3 is a promising candidate therapeutic agent with a novel mode of action that selectively targets the NF-kB survival axis and has significant clinical potential for development in oncology [8]. The safety, tolerability, pharmacokinetics, and pharmacodynamics of DTP3 were recently evaluated in a pilot clinical study in patients with relapsed or refractory MM (first-in-human phase-I/IIa trial, EudraCT: 2015-003459-23), with encouraging initial clinical results [8,9].

Mass spectrometry (MS)-based footprinting and chemical cross-linking (CX-MS) analyses and modeling studies of the GADD45β–MKK7 and MKK7–DTP3 complexes have established that DTP3 interacts with two spatially adjacent MKK7 outer regions, which form a shallow pocket located proximally to the ATP pocket, and that the interactions of GADD45β and DTP3 with MKK7 are mutually exclusive. These data also suggested that the DTP3 binding to MKK7 induces a conformational rearrangement, which contributes to the dissociation of GADD45β from MKK7 [5,11]. GADD45β recognizes the MKK7 catalytic pocket through a flexible acidic loop encompassing residues 103–117. While the 3D crystallographic structure of the kinase domain has been solved [6,12], no structural data are so far available for GADD45β or the complexes GADD45β/MKK7 and DTP3/MKK7 complexes.

In an attempt to further refine at the atomic level the interaction between DTP3 and MKK7 and to shed light on the molecular mechanisms underpinning the effects of DTP3 on the GADD45β/MKK7 interaction, we have investigated the peptide groups that are more directly involved in the binding to MKK7 by using STD-NMR. These analysis were complemented by an extensive computational examination of the interaction mechanisms that could guide future studies aimed at designing novel DTP3-like chemical entities with improved bioavailability and/or drug-like characteristics.

## 2. Materials and Methods

### 2.1. Synthesis and Purification of FITC-$\beta Ala_{2}$-DTP3 and FITC-$\beta Ala_{2}$-SCRB Peptides

DTP3 (Ac-D-Tyr-D-Arg-D-Phe-NH2) and the related scramble (SCRB) peptide (Ac-D-Arg-D-Phe-D-Tyr-NH2) were prepared as previously reported [10]. The fluoresceine isothiocyanate (FITC)-labeled variants (FITC-$\beta Ala_{2}$-DTP3 and FITC-$\beta Ala_{2}$-SCRB) were similarly assembled on the solid phase introducing on the N-terminus two additional β-alanines and then FITC at the N-terminus via direct overnight coupling (room temperature) to the resin. FITC was dissolved in DMF and used at 5-fold excess at slightly basic pH. After removal from the solid support by treatment with a TFA/H${}_{2}$O/tri-isopropylsilane(TIS) mixture at 90:5:5 (*v*/*v*/*v*) (RT, 3 h, 1.0 mL mixture/100 mg resin) and lyophilization, peptides were purified by reversed-phase HPLC using an X-Bridge Prep C18 Column (19 × 150 mm ID) by applying a linear gradient of solvent B (0.1% TFA in CH${}_{3}$CN) over solvent A (0.1% TFA in H${}_{2}$O) from 5% to 70% in 15 min (flow rate 15 mL/min) using a Waters HPLC preparative system. Products purity and identity were assessed by ESI-TOF-MS analyses. The recombinant kinase domain of MKK7 (MKK7-KD), residues 101–405, was prepared as reported elsewhere [5,10].

### 2.2. Fluorescence Quenching Ligand Binding Assay

Fluorescence quenching binding analyses were performed by titrating FITC-$\beta Ala_{2}$-DTP3 and FITC-$\beta Ala_{2}$-SCRB, both at 10 nM, with soluble MKK7-KD at increasing concentrations ranging between 0 and 1000 nM. Experiments were performed in 96-well black plates using 200 μL volume samples in quadruplicates. Excitation was set at 488 nm, while emission spectra were collected between 500 nm and 600 nm. Maximum emission values at 517 nm were averaged and the blank subtracted. $K_{D}S\Delta$fluorescence value were obtained by subtracting the fluorescence signal of the peptide alone plotted against the kinase concentration and then fitted using a nonlinear one-site total algorithm implemented in GraphPad Prism version 5.00 for Windows (GraphPad Software, San Diego, CA, USA). Experiments were repeated at least twice in triplicate. The concentrations of the labeled peptides were determined according to the Lambert–Beer law by using the molar absorptivity of FITC at 488 nm (73,000 cm${}^{-1}$M${}^{-1}$). The very strong emission of FITC prevented the exploration of the wider concentration intervals required to assess the non-specific interaction of the control peptide with the kinase at concentrations above those shown. Experiments were therefore performed by monitoring the tryptophan fluorescence quenching as a function of the concentration of the added peptides. MKK7-KD was used at 1.25 µM in phosphate buffer pH 7.5 together with both DTP3 or SCRB in the concentration range between 10 nM and 140 µM, as previously reported [5]. Experiments were performed at least twice, and data were averaged and plotted against the change in fluorescence emission at 334 nm (ΔFluorescence 334 nm) as a function of the peptide concentrations. To estimate the KD values, data were fitted using GraphPad with a nonlinear algorithm (log[inhibitor] vs. response—Variable slope) to account for the entire concentration range.

### 2.3. NMR Analyses

#### 2.3.1. *NMR Spectroscopy of Free Molecules*

All NMR experiments were performed at T = 301 K by using a Varian Inova spectrometer located at the “Istituto di Biostrutture e Bioimmagini (IBB) of CNR, Napoli”, operating at a proton frequency of 600 MHz, and equipped with a 5 mm inverse-detection cryoprobe and z-gradient. The free MKK7-KD protein was measured by NMR at 10 µM concentration in 600 µL of deuterated TRIS buffer 20 mM/D2O (100%) at pH 7.5, with NaCl 50 mM and TCEP (Tris(2 carboxyethyl)phosphine) 0.5 mM.

One-dimensional and STD spectra of the free MKK7-KD protein were obtained to test the integrity of the protein and to determine appropriate saturation frequencies, respectively. The frequency of −3045 Hz (0 ppm) was chosen as the best one for the magnetization transfer from protein to the peptide binder. Analogously, STD spectra were acquired for each peptide, DTP3, SCRB, and a unrelated control peptide (1 mM in D2O), to verify that they were not excited by the pulse at the frequency chosen for protein saturation. In such a way, the saturation frequency at 0 ppm could be confirmed as the best also for the peptides. The concentration of the unlabelled peptides and protein were spectrophotometrically determined according to the Lambert–Beer law using $\epsilon_{275\mathrm{nm}}$= 1420 cm${}^{-1}$M${}^{-1}$ and $\epsilon_{280\mathrm{nm}}$ = 31,400 cm${}^{-1}$M${}^{-1}$, respectively.

#### 2.3.2. *NMR Spectroscopy of Tripeptide–Protein Interactions*

STD NMR experiments were performed at T = 301 K by adding increasing amounts of peptide to the protein samples at 10 µM in 600 µL of buffered D2O (solvent composition specified above) in order to achieve peptide/protein molar ratios, R, ranging from 10 (R10) to 100 (R100). The excitation sculpting pulse sequences were used to suppress the water signals in the spectra. The protein was irradiated at δ H 0 ppm (on-resonance) and δ H 27 ppm (off-resonance) with a train of Gaussian shaped pulses (50 ms). The broad resonances of the protein were suppressed with a 50 ms spin-lock pulse. The setup of the STD NMR experiments was optimized by a series of experiments using ligand-only samples to ensure that the irradiation at the selected frequency for on-resonance scan did not affect the ligand, as reported above. The saturation time used in the STD experiments was 2 s. Following the method of Mayer and Meyer [13] we also performed a Group Epitope Mapping (GEM) study to identify the binding surfaces on the ligand using STD methods. This approach was based on the comparison of the STD response for different protons within a ligand. This was done by normalizing all the measured STD signals against the one most intense in the spectrum, which is arbitrarily assumed to be the 100% value. The set of resulting STD percentages qualitatively delineates the chemical moieties that are critical for the molecular interaction, as they are intimately recognized by the protein (STD values close to 100%), and the regions of the ligand situated far from the receptor binding site. The proton resonances of the peptides detected in the presence of the protein, assigned at the peptide/protein ratio equal to R100, are reported in Tables S1 and S2 (Supplementary Material).

### 2.4. Computational Studies

#### 2.4.1. *Molecular Dynamics Simulations*

We used the PDB files of the MKK7 and the .mol2 file of the peptides DTP3 and SCRB that were employed in our previous work [5]. SwissParam software was used to generate the topologies and parameters based on the Merck molecular force field, in a functional form that is compatible with the CHARMM force field [14]. After solvation using the SPC water model [15] and minimization procedure performed by the steepest descent algorithm, a 100 ps long NVT and a 100 ps long NPT equilibration runs were performed for each system. Finally, production runs lasting 15 ns for both the peptides and 500 ns for the protein, with a time step of 2 fs, was performed. The Verlet cut-off scheme was adopted and the particle–mesh Ewald method was utilized to treat long-range electrostatic interactions. The temperature was kept constant by means of the velocity rescale algorithm (300 K) [16]. The simulations were executed using Gromacs Software version 2019.4 [17].

#### 2.4.2. *Zernike Descriptors*

First, we calculated the electrostatic potential by assigning to each atom of the system a partial charge as obtained using the PDB2PQR algorithm [18,19]. Given a function f(r,θ,ϕ) describing the molecular surface shape or electrostatics, the Zernike formalism relies on a series expansion in an orthonormal sequence of polynomials:

$$f\left(r,\theta ,\phi \right)\;=\;\sum_{n=0}^{\infty }\sum_{l=0}^{n}\sum_{m=-l}^{l}C_{nlm}Z_{nl}^{m}\left(r,\theta ,\phi \right)$$

where $C_{nlm}$ are the Zernike moments and $Z_{nl}^{m}$ are the Zernike polynomials. The order *N* at which the sum over *n* is truncated selects the level of representation details. In this work, we chose N = 20. The invariant 3D Zernike descriptors are defined as

$$D_{nl}\;=\;||C_{nlm}\left|\right|\;=\;\sqrt{\sum_{m=-l}^{l}{\left(C_{nlm}\right)}^{2}}$$

Selecting N = 20, we deal with 121 descriptors $D_{nl}$. A detailed mathematical treatment is reported in Venkatraman et al. [20].

In order to characterize the binding properties of the interfaces between two interacting molecules, we considered both their shape and electrostatic complementarity. As both binding regions can be correctly described with a set of Zernike descriptors, we were able to quantify the degree of complementarity (DOC) between the two interfaces measuring the distance between the two vectors of Zernike descriptors. In particular, the higher was the shape complementarity between the two interacting regions, the shorter was the distance between the corresponding Zernike descriptors (i.e., similar surfaces have similar descriptors). In order to analyze the role of the electrostatics in the binding process, we compare, in terms of the Zernike descriptors, the surface corresponding to the positive electrostatic potential with the surface of the negative electrostatic potential (and vice versa). This approach is justified since the positive potential descriptors of one region has to be similar to the negative potential descriptors of the interacting region (and vice versa). Therefore, the complementarity between regions A and B is defined as

$$\begin{array}{c} \;{\left[A-B\right]}_{shape}\;=\;D\left(X_{shape}^{A},X_{shape}^{B}\right)\\ \;{\left[A-B\right]}_{elec}\;=\;\frac{(D\left(X_{elec,+}^{A},X_{elec,-}^{B}\right)+\left(D\left(X_{elec,-}^{A},X_{elec,+}^{B}\right)\right)}{2} \end{array}$$

where $X_{shape}$, $X_{elec,+}$, and $X_{elec,-}$ are the shape, the electrostatic positive, and the electrostatic negative descriptors, respectively. D represents the cosine distance. Note that high complementarity is achieved when these distances are small.

#### 2.4.3. *Binding Sites Detection and Molecular Docking*

DTP3 binding sites on MKK7 protein were predicted by using the P2Rank approach, a template-free, machine learning-based method for ligand binding site prediction which uses a random forest model to predict “ligandability” scores for each point on a protein’s surface [21]. Cluster points with high scores into resulting pocket scores are used to rank the putative pockets. AutoDock Vina (version 1.1.2) was used in this study to perform docking experiments [22]. We used the same PDB files of the MKK7 (receptor) and the .mol2 file of the peptides DTP3 and SCRB (ligands) that were used in our previous work [5]. The parameters were set employing AutoDock Tools [22], and the protein structural files were translated to PDBQT (Protein Data Bank, Partial Charge (Q), and Atom Type (T)) format by adding polar hydrogens and Kollman charges in the same software. All rotatable bonds within the peptides (ligands) were allowed to rotate freely. One of the critical parameters for ligand docking is the size of a search space used to identify low-energy binding poses of ligand candidates. The docking search space was defined according to the procedure for calculating the optimal docking box size that maximizes the accuracy of binding pose prediction described by Feinstein et al. [23]. Autodock Vina software provides a parameter called “Exhaustiveness” to change the amount of computational effort used during a docking experiment. The default exhaustiveness value is 8, but we increased the exhaustiveness of search parameter to the value of 256 (the number of final binding modes produced by Vina was set to 20) in order to give a more consistent docking result. All other docking parameters were set to the default values. Subsequently, each of the conformation was visualized on UCSF Chimera, and the best docked orientation was selected based on docking score and binding interactions with the protein. The hydrogen bond (HB) and hydrophobic (HP) contacts between ligand and receptor were estimated using the protein-ligand interaction profiler LigPlot+ [24].

## 3. Results

### 3.1. Peptide Synthesis

DTP3, SCRB, and the FITC-peptides were obtained with average yields of 90% and after RP-HPLC purification homogeneous products were isolated. Experimental molecular masses were consistent with the expected values (DTP3 and SCRB: MWtheor 526.20 amu/MWexp 526.18 amu. FITC-$\beta Ala_{2}$-DTP3 and FITC-$\beta Ala_{2}$-SCRB: MWtheor 944.16 amu/MWexp 943.37 amu).

### 3.2. Fluorescence Quenching Ligand Binding Assay

**Figure 2:**
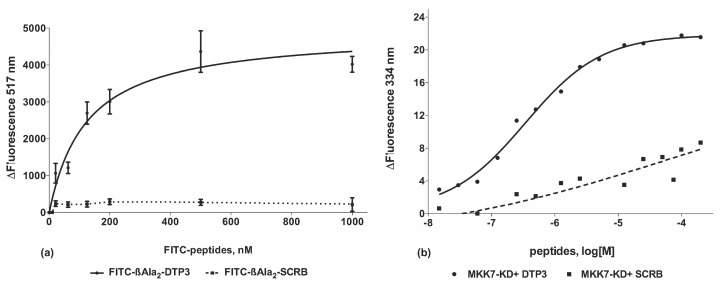
Fluorescence quenching binding analysis of N-terminally fluoresceinated DTP3 to MKK7-KD. (**a**) ΔFluorescence values at 517 nm of FITC-$\beta Ala_{2}$-DTP3 and FITC-$\beta Ala_{2}$-SCRB (10 nM) plotted against the concentration of MKK7-KD (0.05 ÷ 1000 nM) are reported. Values denote means ± SD (n = 4). (**b**) Fluorescence quenching binding analysis of DTP3 and SCRB to MKK7-KD as obtained by monitoring the ΔFluorescence at 334 nm. ΔFluorescence values plotted against the concentration of peptides (10 nM ÷ 140 µM) are reported. Values denote means ± SE (n = 2).

According to previously reported results, obtained by similar experiments [5], the binding affinity of DTP3 (tested as FITC-$\beta Ala_{2}$-DTP3) to MKK7-KD was in the nanomolar range (KD = 123.2 ± 35.3 nM). DTP3/MKK7-KD binding was assessed evaluating the fluorescence quenching of the FITC molecule, covalently bound to the DTP3 N-terminus, that we obtain as the peptide is located in proximity to the kinase. In a parallel experiment performed with the control peptide FITC-$\beta Ala_{2}$-SCRB, no such quenching was observed, indicating that the binding was due to the tripeptide and not the N-terminal FITC molecule (See Figure 2a). The fluorescence quenching binding curves obtained by titrating DTP3 and SCRB in the range of concentrations between 10 nM and 140 µM with the kinase domain of MKK7 are reported in Figure 2b. Under these conditions, DTP3 bound to MKK7-KD with a KD of 0.240 ± 0.070 µM in substantial agreement with the previous experiment using an alternative methodology. Titration of the SCRB peptide with MKK7-KD instead only showed some binding at concentrations above 10 µM (with a ΔFluorescence at 334 nm of about 4 units) and was not saturated up to 140 µM. Fitting of the SCRB data with the same algorithm provided a KD of around 41 µM.

### 3.3. Interaction studies of DTP3 and SCRB with MKK7-KD by STD-NMR

**Figure 3:**
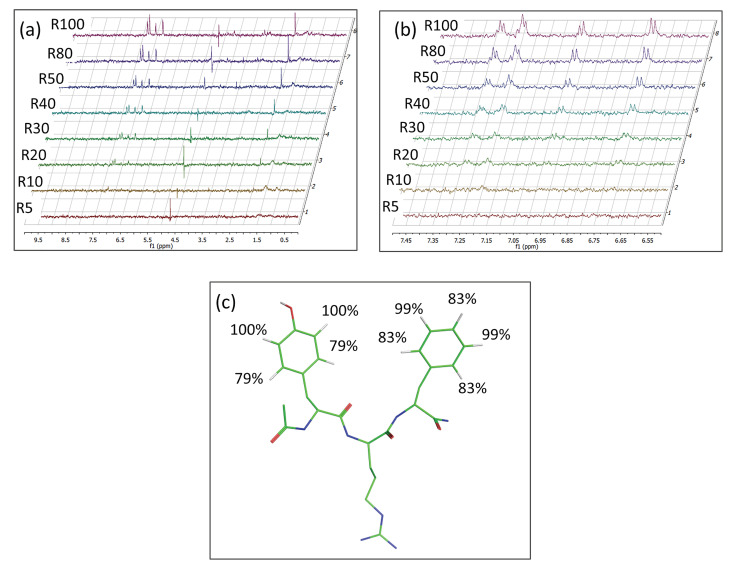
(**a**) STD spectra acquired at 10 µM of MKK7-KD and DTP3/MKK7-KD ratio values in the range 5 ÷ 100; (**b**) Zoom-in of the aromatic region; (**c**) Molecular model of DTP3 reporting the group epitope mapping (GEM) at R100.

Using a STD-NMR technique, we investigated the interaction of MKK7-KD with DTP3, SCRB, and other unrelated peptides. For each peptide, a series of STD spectra was acquired by using a fixed protein concentration of 10 µM and increasing peptide/protein R ratios. The full STD spectra together with expansions of the aromatic region, obtained for DTP3/MKK7-KD at ratio values from 5 to 100, are shown in Figure 3a,b. While for R = 5 no signals were visible, STD signals from D-Tyr${}^{1}$ and D-Phe${}^{3}$ aromatic protons appear starting from R = 10. Signals due to βCH and βCH2 protons of D-Arg${}^{2}$ and acetyl group were observed only starting from R = 50. A comparison of the 1D and STD spectra is shown in Figure S1 (Supplementary material). At each R value, different nuclei are more or less affected by magnetization transfer in reason of their closeness to the enzyme surface during the contact events. A picture of the binding epitope of the ligand can be obtained by Group Epitope Mapping (GEM) [13], that is by comparing the STD effects of the protons once normalized with respect to the highest STD response. The set of normalized STD values qualitatively describes the portions of the ligand that are most involved in the interaction with the target protein. The GEM analysis performed for DTP3 (at R = 100) shows that the aromatic side chains are the most important for the interaction with MKK7-KD (Figure 3c). Taken together with the lower effects measured for the central residue, this result suggests that the most productive DTP3 conformation for MKK7 binding requires an iso-orientation of D-Tyr${}^{1}$ and D-Phe${}^{3}$ aromatic rings and an opposite localization of the D-Arg${}^{2}$ side chain.

**Figure 4:**
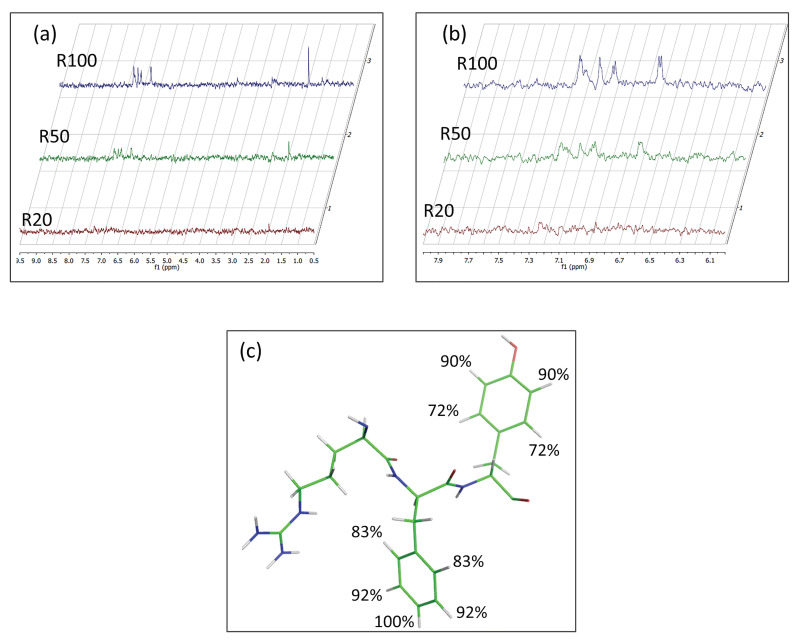
(**a**) STD spectra acquired at 10 µM of MKK7-KD and SCRB/MKK7-KD ratio values in the range 5 ÷ 100; (**b**) Zoom-in of the aromatic region; (**c**) Molecular model of SCRB reporting the group epitope mapping (GEM) at R100.

A GEM analysis of the binding surface of SCRB evaluated at R = 100 shows that, as for DTP3, the aromatic side chains are the moieties most involved in the MKK7-KD interaction (Figure 4c). A comparison of 1D and STD spectra is reported in Figure S2 in the Supplementary Material. At the concentrations used for STD analysis, DTP3 and SCRB peptides are both able to interact with the enzyme even though with different affinities (Figure 4). Indeed, the STD comparison demonstrates that the presence and the arrangement of the two aromatic residues do matter, with the distanced arrangement (i and i + 2 positions) that are more effective than the sequential one (i and i + 1). The saturation transfer effect also observed with the SCRB peptide likely derives from the high concentrations of the peptide necessarily required for the NMR STD experiments. However, it is apparent from the comparison spectra shown in Figure S3 that the STD effect observed with DTP3 can be recorded, yet at R20, it is almost absent in the parallel experiment with SCRB. Moreover, the STD intensities recorded in the parallel experiments conducted at grater concentration ratios are higher for DTP3 than for SCRB, suggesting a stronger interaction of MKK7 with DTP3 than with SCRB. Potentially owing to the structure similarity of the two isomeric tripeptides, at concentrations above 10 µM, the SCRB peptide also showed some binding with the kinase, consistent with results from the fluorescence quenching experiments (see Figure 2b). However, experiments performed at more biologically relevant concentrations demonstrated that DTP3, but not SCRB was capable of binding to MKK7 (See Figure 2, see also Tornatore et al. [5] and Rega et al. [11]). To confirm the specificity of DTP3 for MKK7-KD, we investigated the potential interactions with additional peptides having a similar size but no aromatic side chains. The set of STD spectra acquired under similar conditions with one of these control peptides [25] is reported in Figure S4 (Supplementary Material), which shows no binding to MKK7-KD at any of the concentrations tested.

### 3.4. Identification of the DTP3 Binding Sites on MKK7-KD Surface and Their Characterization by Using Zernike Polynomials

While we previously identified the petide segments of MKK7-KD involved in the interaction with DTP3 (Y113-K136 and L259-K274, according to UNIPROT numbering) [11], the molecular details of the 3D kinase binding site and modality of the DTP3-dependent interaction remain unknown.

**Figure 5:**
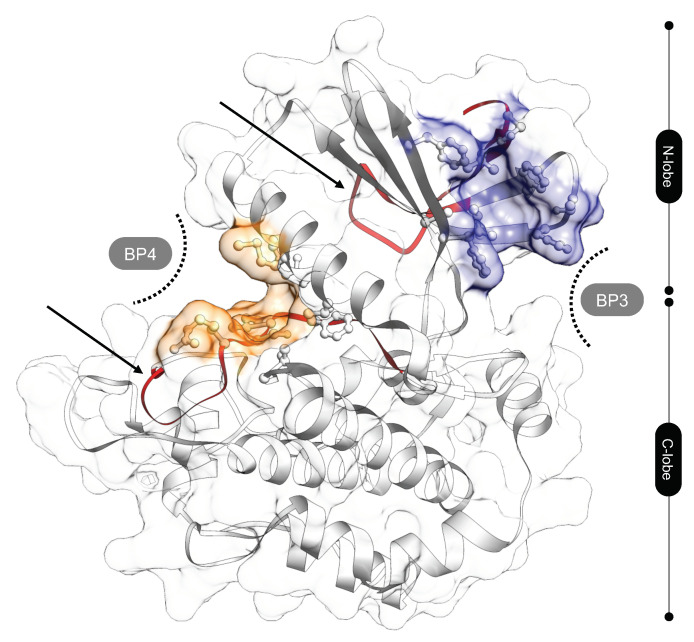
BP3 (in blue) and BP4 (in orange) binding sites on the kinase domain surface of MKK7, potentially occupable by DTP3. MKK-KD is shown in cartoon and surface representations. The residues defining the binding site are depicted in ball and stick representation. MKK7 linear peptides involved in the interaction with DTP3 [11] are reported as red ribbons (black arrows, residues 113–136 and 259–274).

To identify the DTP3 binding sites of MKK7, we first used the P2Rank software. P2Rank scanned the surface and identified four top ranked binding pockets, hereafter defined as BP1, BP2, BP3, and BP4, that are potentially involved in the interaction with the DTP3 peptide. BP3 and BP4 pockets (Figure 5) were selected for further evaluation because they are in complete agreement with previous predictions [5] and experimental data [11]. In particular, several residues of BP3 and BP4 were previously identified as being involved in the interaction with DTP3 [11] (depicted as red ribbons in Figure 5). Of note, BP1 corresponds to the ATP binding site, which was previously reported not to be involved in the DTP3 interaction [11]. BP3 lies within the N-lobe of the MKK7-KD, while BP4 is located in the cleft between the N- and C-terminal lobes. BP3 and BP4 are defined by the residues 119, 120, 139, 142, 144, 146, 183, 186, and 195, and 162, 165, 166, 169, 239, 262, 263, 264, 265, 266, and 267, respectively (Figure 5).

We subsequently investigated the interaction between DTP3 with both BP3 and BP4 by using a method dealing with series expansion of the function that represents the protein molecular surface based on the Zernike polynomials. The norm of the expansion coefficients provides a local description of the shape and the electrostatic configuration of the regions involved in the interaction [20,26,27]. Once the molecular surfaces are described with the Zernike descriptors, complementarity metrics for both the shape and the electrostatics can be easily defined using a pairwise distance (we adopted the Cosine distance). It results that the lower the distance between their Zernike descriptors, the higher the complementarity between the two potentially interacting molecular regions (See Experimental Section) [28,29]. By such an approach, we can quantitatively assess the complementarity between two molecular regions, belonging to different molecular partners, that are supposed to interact. To let the peptides and the protein explore their own conformational spaces at equilibrium and to obtain a more statistically robust result, we performed Molecular Dynamics (MD) simulations to get representative snapshots of the structures of the molecules. In particular, we matched, by means of Zernike descriptors, the 60 conformations of both DTP3 and SCRB, obtained using a 15 ns long MD simulation performed in water for each peptide, with the 500 BP3 and BP4 conformations obtained from 500 ns long MD simulation of MKK7-KD in water. Before going further, MKK7 stability and its conformational behavior were investigated. We studied the backbone RMSD and gyration radius evolution over the MD trajectory, revealing how the initial 30 ns are required for MKK7 equilibration. Then, a reliable structural stability was reached and the protein does not destabilize the global conformation during the simulation (Supplementary Figures S5 and S6a). We also evaluated the local conformational mobility of the regions potentially interacting with DTP3, BP3, and BP4, by means of studying RMSF parameter and how the volume of BPs changes during the MD simulation. Results of this analysis, reported in Figure S7, showed that the BP4 pocket residues do not display great displacement since the corresponding RMSF values are low for each residue. The BP3 cavity is characterized by higher atomic mobility during molecular dynamics (especially residues 119 and 142), but it is not the region of the protein with the greatest structural variation. Moreover, BP3 is partially composed by the last N-terminal region of MKK7 and this may explain its higher mobility. In fact, even BP3 volume variation analysis did not display any marked variation during MD simulation (Figure S7b) Then, we evaluated the shape and electrostatic complementarity between the peptides and the two pockets on the MKK7-KD surface.

**Figure 6:**
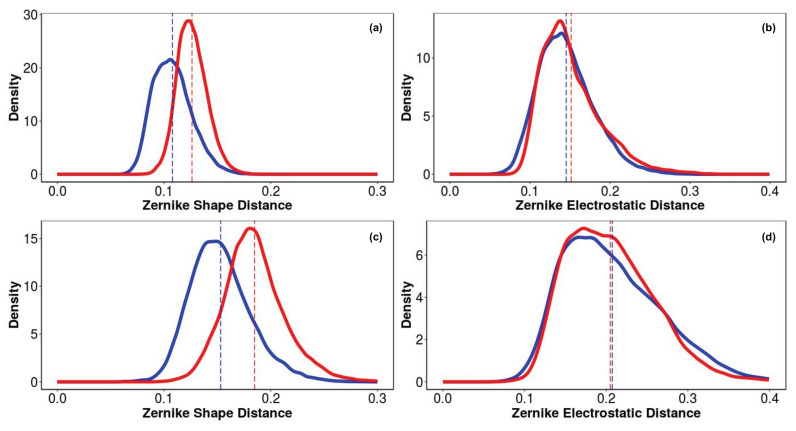
Density distribution of Zernike descriptors calculated for DTP3 and SCRB are reported. Shape and electrostatic complementarity between DTP3 (blue lines) and SCRB (red lines) peptides with BP3 (**a**,**b**) and BP4 (**c**,**d**) pockets of the MKK7 surface.

The Zernike descriptors of shape and electrostatics complementarity, obtained as results of this analysis, are depicted in Figure 6a–d. It is worth noting that shape complementarity is a distinguishing feature of the binding properties of DTP3 and SCRB, and likely drives the specific interaction of DTP3, but not SCRB, with both BP3 and BP4 (Figure 6a,c, respectively). The density distribution difference of the Zernike distances is statistically significant (KS-test *p*-value is less than 2.2 × 10 ${}^{-16}$). In particular, DTP3 has a lower Zernike shape distance than SCRB in both the BP3 and BP4 pockets, suggesting a higher shape complementarity. On the other hand, the electrostatic interactions do not seem to play a role in the selective binding of DTP3 relative to SCRB to BPs. Indeed, the DTP3 and SCRB Zernike electrostatic distance density distributions are substantially overlapped. (Figure 6b,d).

The shape analysis also reveals that the Zernike distances estimated for the BP3 cavity, positioned at the N-terminus of MKK7, are lower compared to that exhibited by BP4 (Figure 6c) regardless of the peptide involved. These findings suggests an overall better predisposition of BP3, in terms of shape complementarity, to the interaction with these peptides in all their possible conformations.

Starting from the STD data and considering the atomic structures DTP3 and SCRB, we also investigated, through the procedure based on the Zernike formalism, the changes in the complementarities between the binding site of the protein and the corresponding interacting peptide. In particular, we analyzed, comparatively for the two peptides, the distribution of the spatial distances between the centroids of the side chain (geometric center of the heavy atoms) of the aromatic residues during the thermal motion in water as calculated by Molecular Dynamics (MD) simulations. This measure allows us to characterize the different conformations of the peptides, recognizing more open conformations from more compact ones.

**Figure 7:**
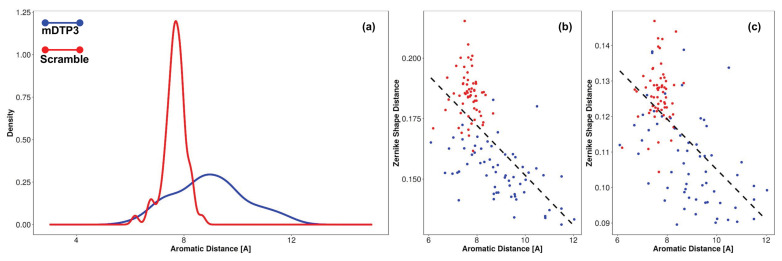
(**a**) Distribution of spatial distances between side chain heavy atoms geometrical center of aromatic residues of DTP3 and SCRB peptides. (**b**,**c**) Correlation of the distances explored by the peptides with shape complementarity to the binding pockets BP3 and BP4, respectively.

We also evaluated the correlation between the explored distances with shape complementarity, measured for the putative binding pockets (BP3 and BP4). From these analyses, we found that the aromatic residues of DTP3 (position i and i + 2) explore a very wide range of distances (Figure 7a, blue line), while the distance between the aromatic rings of SCRB (i and i + 1) is on average lower than that observed with DTP3 (Figure 7a, red line). A relevant result for clarifying the molecular mechanisms of the DTP3 binding to MKK7, is the anticorrelation between the aromatic ring distances of the DTP3 peptide with the Zernike shape distance (Figure 7b,c, blue dots). Aromatic distances have a Pearson correlation coefficient value of −0.64 and −0.58 with BS4 and BS3, respectively (*p*-value less than 10${}^{-5}$). SCRB peptide aromatic distances demonstrate instead no correlation with the Zernike shape distance (Figure 7b,c, red dots). These results clearly demonstrate that the complementarity of DTP3 with BP3 and BP4 is mainly mediated by the possibility of this molecule to increase the distance between the two aromatic rings. In particular, we found that the further the aromatic residues are, the higher the complementary of the peptides with the pockets is. On the other hand, having an almost fixed “aromatic distance”, the SCRB peptide cannot achieve the same complementarity for BP3 and BP4 as DTP3.

### 3.5. MKK7-DTP3 Molecular Docking Studies

Subsequently, we obtained a more accurate picture of the binding modalities of DTP3 with BP3 and BP4 by means of a focused molecular docking study. These two cavities were defined in the docking experiment using the residues identified in the previous section, and the docking analyses were performed using the Autodock Vina program. Hydrogen bond (HB) and hydrophobic (HP) contacts between DTP3 and MKK7-KD were estimated using LigPlot+ software [24].

**Figure 8:**
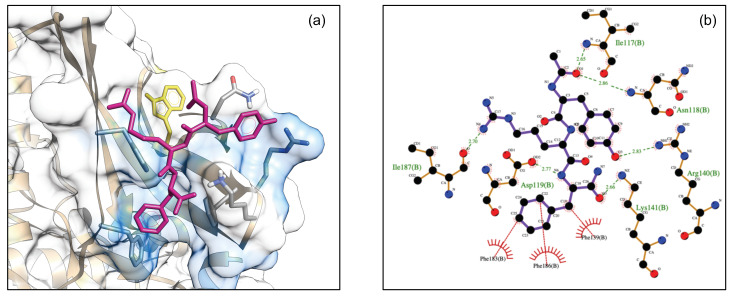
(**a**) Surface model of DTP3 docked to BP3 (cyan) where the protein molecule is shown as surface and DTP3 is shown in stick (magenta). Trp135 located in proximity of the DTP3 binding site is depicted as yellow sticks; (**b**) Interactions of the MKK-KD residues with DTP3 interactions of the MKK-KD residues with DTP3 are displayed in a two dimensional model. The main interactions between the protein and the ligands are shown as dotted lines, hydrogen bonds are in green, while the hydrophobic interactions are shown in red.

Figure 8a shows the best docked conformation (top-ranked pose) of DTP3 with the BP3 cavity of MKK7-KD (ΔG = −11.47 kcal/mol). The 2D ligand interaction diagram of DTP3 is shown in Figure 8b.

This top-ranked pose suggests a positioning of the tripeptide that is able to make contacts with several protein residues and to adopt an iso-orientation of the D-Tyr${}^{1}$ and D-Phe${}^{3}$ aromatic rings and an opposite localization of the D-Arg${}^{3}$ side chain. This is consistent with results from the NMR-STD experiments, although the NMR data were not used as restraints in the docking run. These results are also consistent with Zernike analysis which confirms that the conformation and the relative orientations adopted by D-Tyr${}^{1}$ and D-Phe${}^{3}$ residues are crucial for the interaction of DTP3 with the MKK7 protein. The residues of the kinase domain involved in hydrogen bonds and hydrophobic interactions with a specific portion of DTP3 are shown in Figure 8b.

In particular, D-Tyr${}^{1}$ formed two H-bonds with Arg140. The phenyl group of D-Phe${}^{3}$ appears to be embedded in a cavity formed primarily by residues Phe139, Phe183, and Phe186. In this conformation, D-Phe${}^{3}$ may form a potential π-stacking with the β2 Phe139. D-Arg${}^{2}$ appears to form two H-bonds with the Ile187 backbone oxygen, while the DTP3 backbone is involved in two H-bonds with Asn118 and Asp119. Taken together, the experimental and computational data point toward the presence of a well-defined and previously unknown binding site in the N-terminal region of MKK7 that is explored by DTP3 molecule. This site, which was found to exhibit allosteric dynamics [5,11], is likely involved in the modulation of the protein-protein interactions with GADD45β [11]. The presence of DTP3 around this N-terminal allosteric site is also consistent with the FITC-tripeptide fluorescence quenching effect induced by the β2 Trp135 side chain [11] located in proximity of the putative DTP3 binding site (Figure 7a).

**Figure 9:**
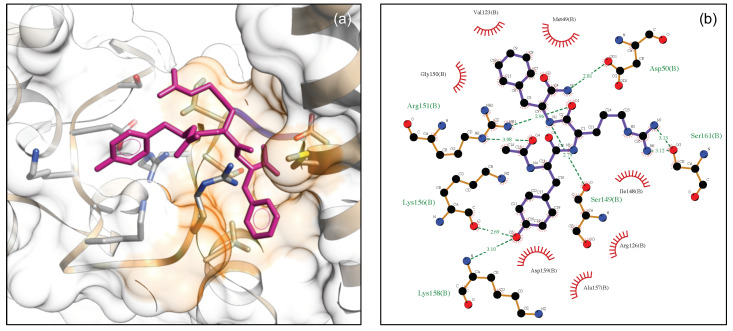
(**a**) Surface model of DTP3 docked to BP4 cavity (orange) where the protein molecule is shown as surface and DTP3 is shown in stick (magenta); (**b**) Interactions of the MKK-KD residues with DTP3 are displayed in a two dimensional model. The main interactions between the protein and the ligands are shown as dotted lines, hydrogen bonds are in green, while the hydrophobic interactions are shown in red.

We also subjected DTP3 to molecular docking at the BP4 site of MKK7 that is located between the αC helix, the activation loop and the D-F-G motif (see Figure 5). The generated docked complexes were examined based on binding affinity values (Kcal/mol) and bonding interaction patterns. The best pose (ΔG = −9.22 kcal/mol according to Autodock Vina program), is reported in Figure 9a. Considering the results, the DTP3/BP4 interface is stabilized by several van der Waals interactions and by a series of intermolecular hydrogen bonds (Figure 9b). Of particular note was the binding mode of DTP3 where the moiety protruded into a hydrophobic pocket in proximity to the αC helix, formed by residues Met 49, Val 123, Ile 148, and Ala 157. This conformation assumed by DTP3 in proximity to the MKK7 surface could destabilize the D–F–G protein motif thus resulting in a MKK conformation alteration.

## 4. Discussion

We have investigated the interaction of DTP3 with the kinase domain of MKK7, the therapeutic target, which is relieved from GADD45β-mediated inhibition upon binding to DTP3, with activation of JNK-dependent apoptosis. Our NMR binding data support the view that DTP3 predominantly interacts with the protein kinase domain via its aromatic side chains and that its arginine residue has a less relevant role in the binding to MKK7. A detailed computational investigation performed by molecular dynamics, prediction of binding sites and molecular docking studies has further elucidated the localization of the best putative MKK7-KD binding site of DPT3 and provided an atomistic view of the interactions established with the protein. A Zernicke analysis has also clarified the nature of the MKK7/DTP3 interactions and provided a basis for the selectivity of DTP3 as compared to the control scrambled (SCRB) peptide. The NMR-STD study has provided information about the peptide surface involved in the interaction with MKK7. Parallel experiments suggested that high, non-biologically relevant concentrations of the SCRB peptide can also force an interaction with MKK7. However, this interaction is lost at concentrations in the therapeutic nanomolar range. Consistently, the STD technique works with dimensionally different molecule pairs that interact with KD values in the millimolar to low micromolar range (10${}^{-3}$–10${}^{-8}$ M), ensuring a proper exchange between the bound and free ligand states that originate the STD signal. Thus, given the NMR technique experimental limitations, it is possible that these experiments only provide a partial view of the molecular MKK7/DTP3 complex and the peptide interacting atoms. Similarly, on the basis of previous data with other control D-tripeptides [5], it is possible that the central arginine also plays a role in the interaction, contributing to the higher affinity measured by fluorescence. The STD intensities of the DTP3 protons demonstrate that the aromatic side chains of D-Tyr${}^{1}$ and D-Phe${}^{3}$ come into contact with the MKK7 surface closer to the side chain of the central residue D-Arg${}^{2}$. The Zernike analysis further suggests that the distance between the two aromatic side chains is critical for an efficient recognition. Thus, altogether, the data suggest that the hydrophobic interactions mediated by the phenylalanine and tyrosine rings play a main role in the docking of DTP3 to MKK7 and possibly in the allosteric effect leading to the dissociation of GADD45β from MKK7.

## 5. Conclusions

The molecular complex formed by MKK7 and GADD45β has been identified as a key therapeutic target downstream of NF-kB in MM. DTP3 effectively blocks the GADD45β/MKK7 interaction, thereby restoring MKK7 kinase activity, and as such is a promising candidate therapeutic for clinical development in oncology. Here, we investigated the interaction of DTP3 with the kinase domain of MKK7 and further elucidated the main molecular determinants underpinning this interaction. We have identified the key hydrophobic groups involved in the MKK7/DTP3 binding and clarified the peptide geometries and main kinase binding pockets, thus advancing the understanding of the mechanism of recognition. Together, the experimental data from our NMR, fluorescence, and computational studies point toward an interaction of the tripeptide with the protein N-terminal region, in agreement with previous observations, and corroborate the hypothesis of a dynamic interaction that ultimately leads to dissociation of the GADD45β/MKK7 complex. These findings improve the understanding of the interactions between these molecules and provide further molecular details for the generation of novel MKK7/GADD45β inhibitors.

## Data Availability

The data presented in this study are available on request from the corresponding author.

## Supplementary Materials

The following are available online at https://www.mdpi.com/2227-9059/9/1/20/s1. Figure S1: Comparison of 1D spectrum of DTP3 with STD of DTP3/MKK7-KD. Figure S2: Comparison of 1D spectrum of SCRB peptide with STD of SCRB/MKK7-KD. Figure S3: Comparison of STD full spectra acquired at R20 of DTP3 and SCRB vs MKK7-KD. Figure S4: STD spectra at different peptide/protein R ratios of peptide used as negative control. Figure S5: Evolution of MKK7 gyration radius over the MD trajectory. Figure S6: (a) Root Mean Square Deviation (RMSD) values assumed by the 500 structures of MKK7 selected from the MD simulation (b) Principal Component Analysis of the 500 MKK7 frames employing protein alpha carbon atoms. Figure S7: (a) Analysis of local conformational mobility of the BP3 and BP4. Root Mean Square Fluctuation (RMSF) values are reported. (b) BP3 cavity volume evolution during the simulation. Table S1: Chemical shifts of DTP3 at peptide/KD R100 molar ratio. Table S2: Chemical shifts of SCRB at peptide/KD R100 molar ratio.
