# Supplementary material for: Insights into the Interaction Mechanism of DTP3 with MKK7 by Using STD-NMR and Computational Approaches
Source: Biomedicines. 2020 Dec 30;9(1):20. doi: 10.3390/biomedicines9010020 (PMC7824710; doi:10.3390/biomedicines9010020)
Supplement: Supplementary file 1 [file biomedicines-09-00020-s001.pdf]

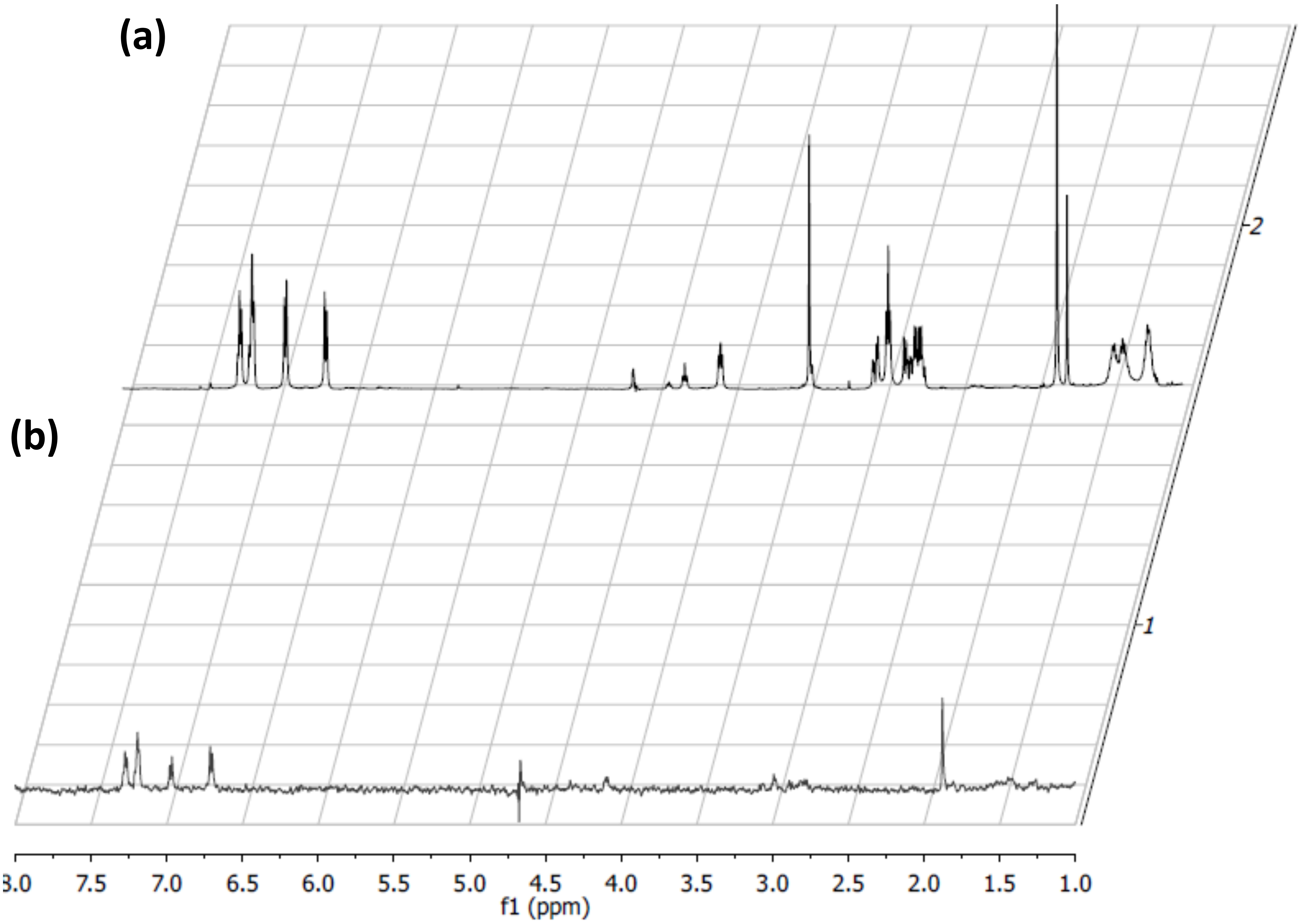

Figure S1: Comparison of 1D spectrum of DTP3 (a) and STD at R100 of DTP3/MKK7-KD (b)

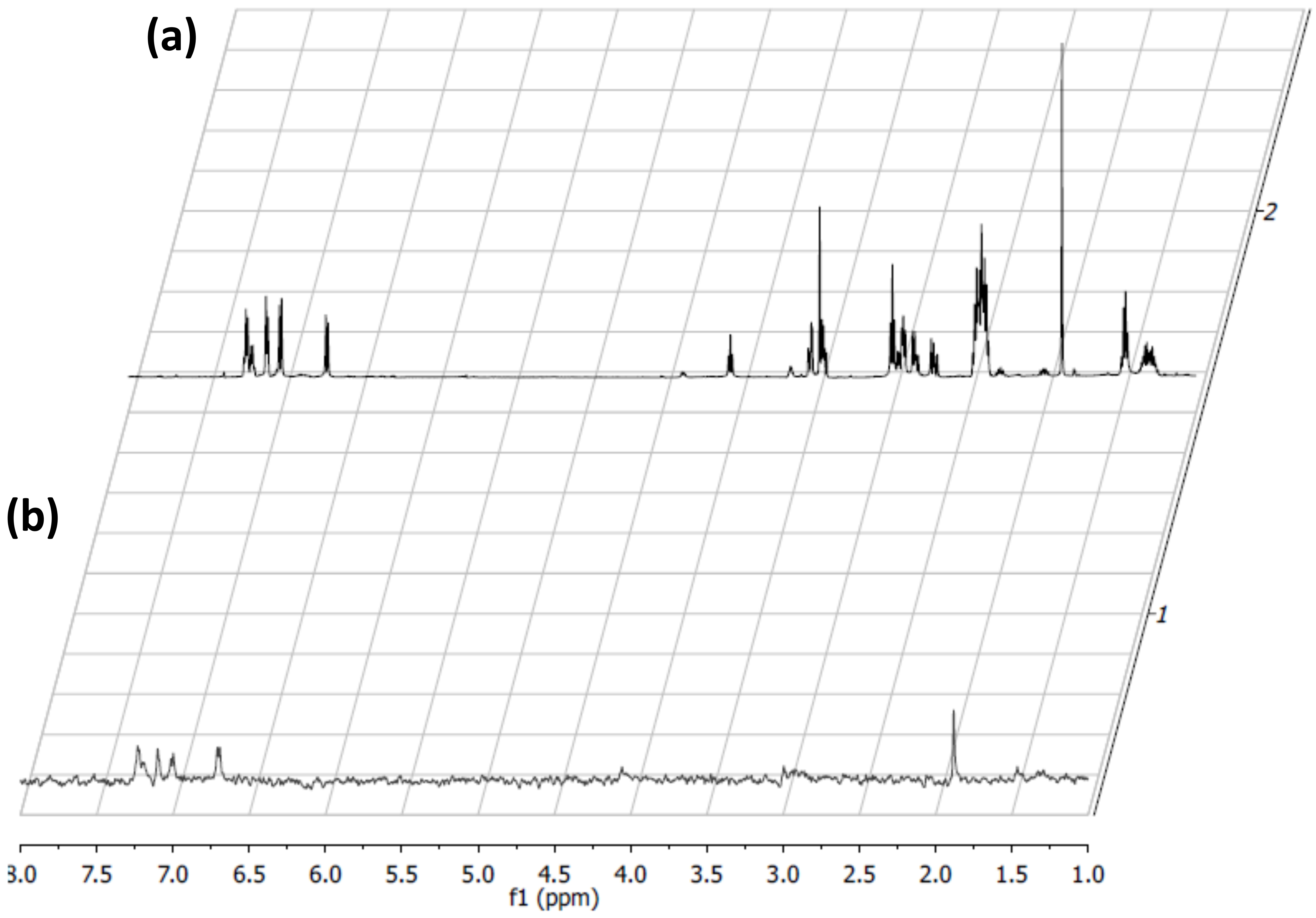

Figure S2 Comparison of 1D spectrum of SCRB (a) and STD at R100 of SCRB/MKK7-KD (b)

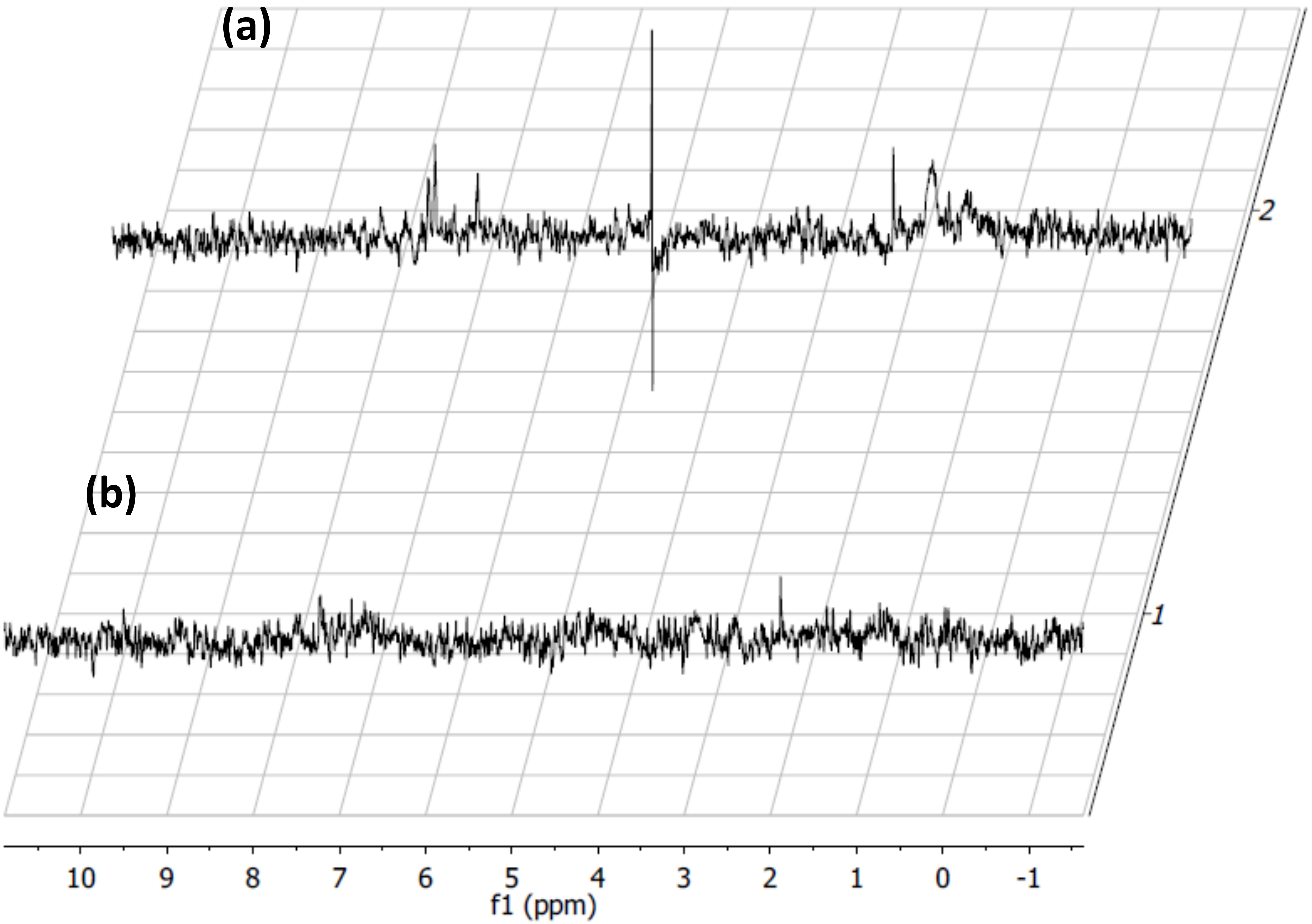

Figure S3 Comparison of STD full spectra acquired at R20 of DTP3 (a) and SCRB (b) vs. MKK7-KD

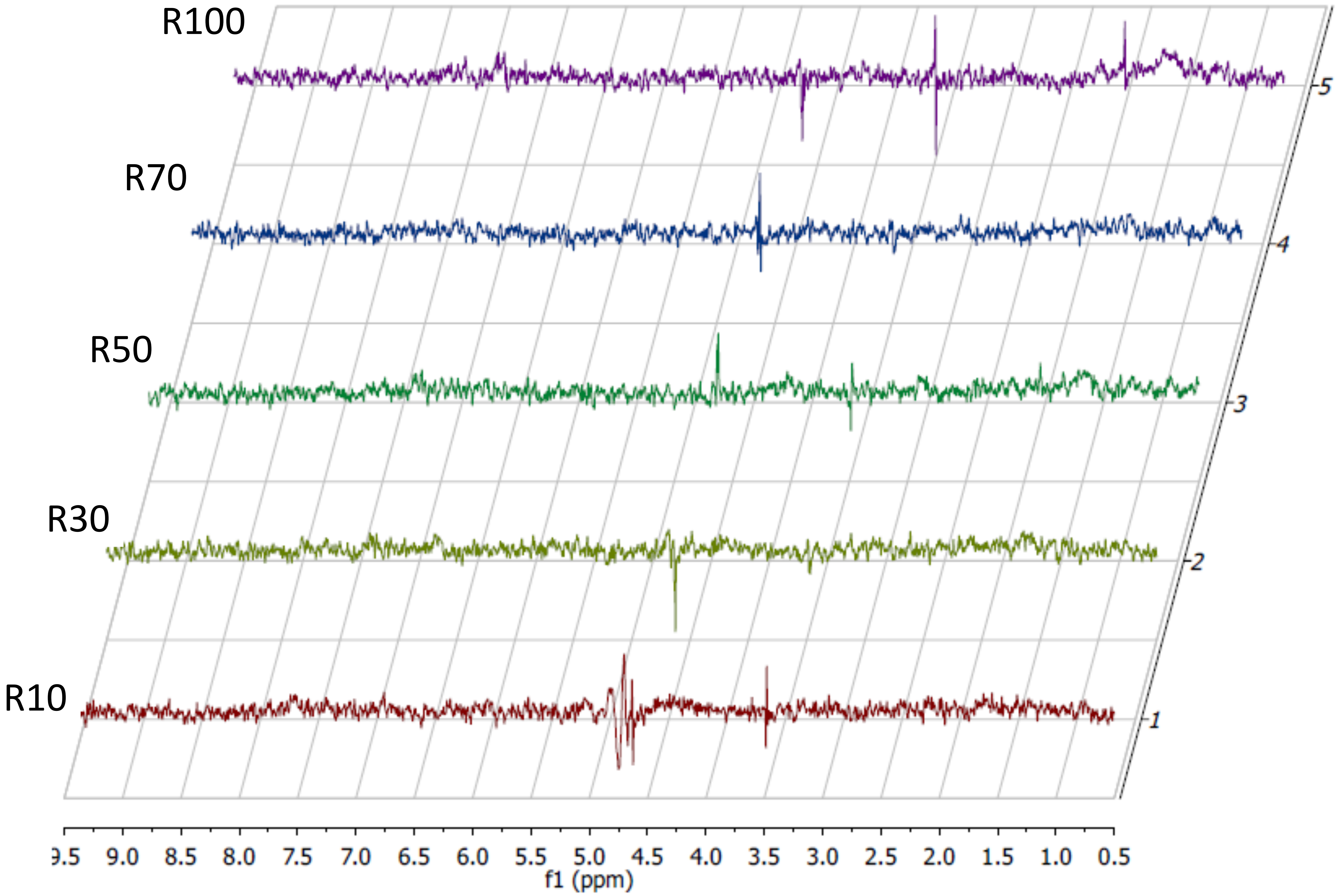

Figure S4: STD spectra at different peptide/protein R ratios of CH3CO-D-Tyr1-D-Glu2-D-Arg3-D-Phe4-NH2 used as negative control

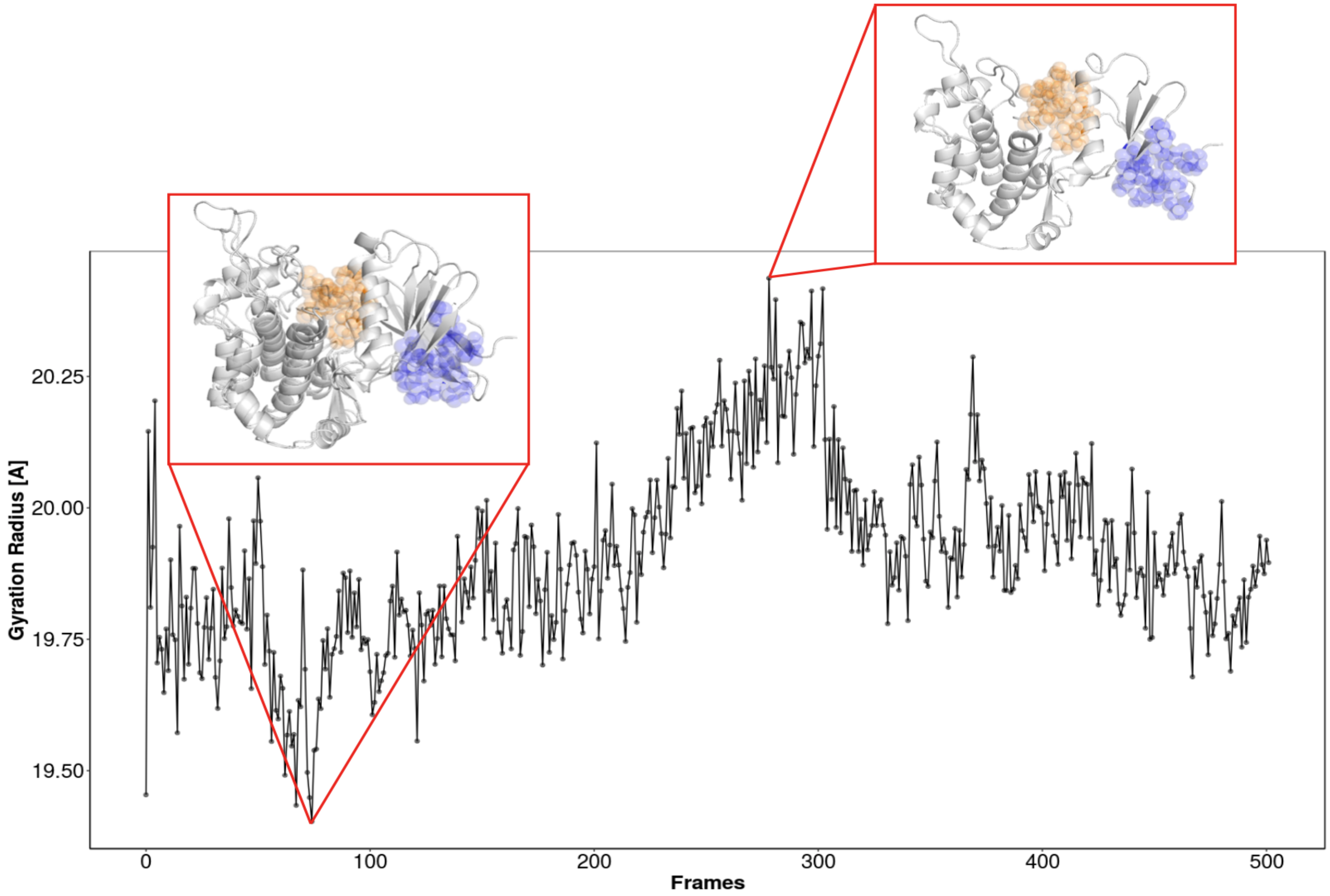

Figure S5: evolution of MKK7 gyration radius over the MD trajectory. In the figure are reported values obtained analysing all the 500 simulation frames: the difference between the highest and the lowest value is 1.03Å, indicating that the protein does not destabilize the global conformation during the simulation frames explored. To better visualize the conservation of the BP3 and BP4 conformation, we also reported the representation of the two most different structures, indicating the shape of the two cavities.

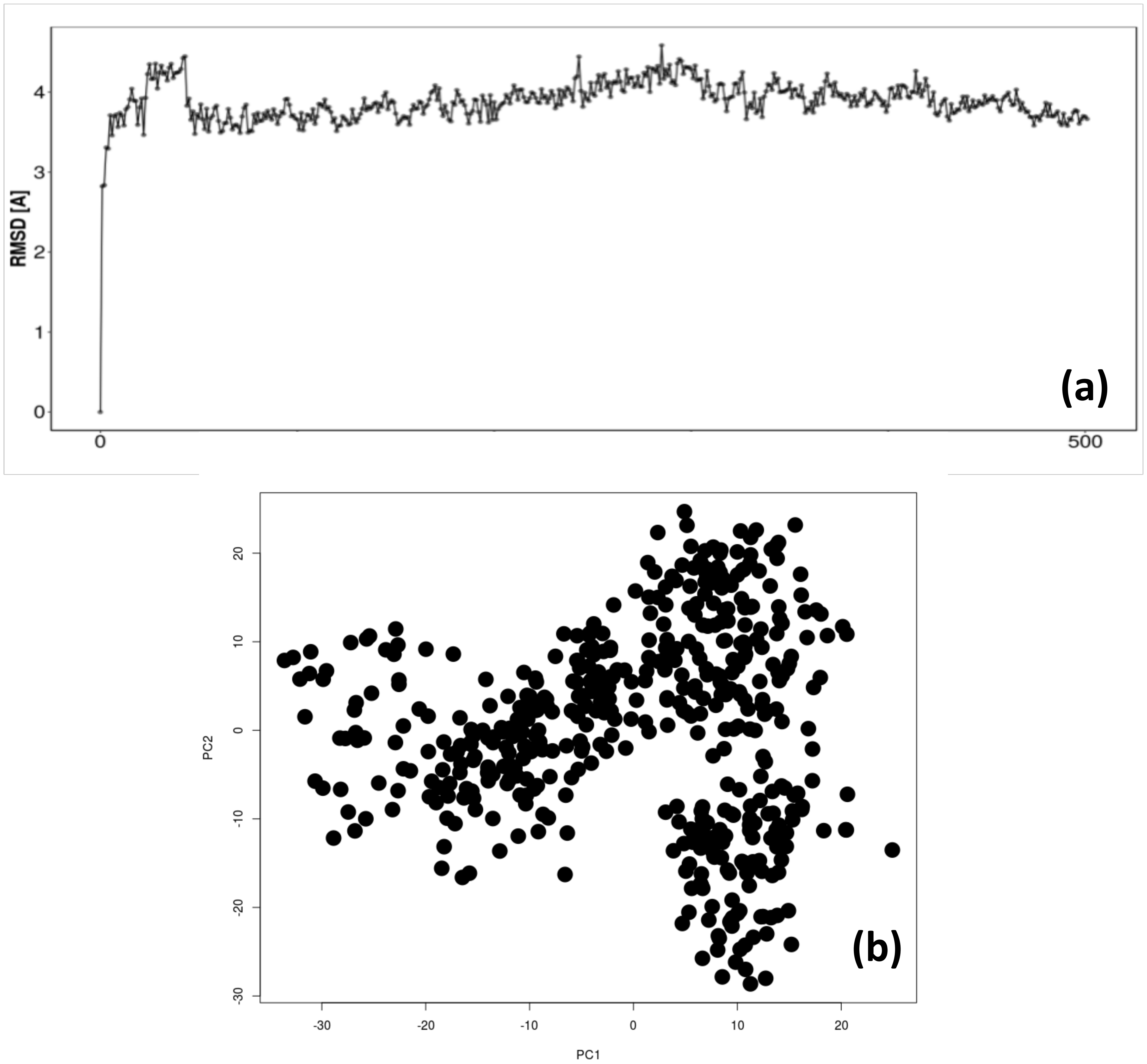

Figure S6: (a) Root Mean Square Deviation (RMSD) values assumed by the 500 structures of MKK7 selected from the MD simulation. The protein reaches the equilibrium state within 30 ns simulations. We divide the frames into three equal parts and we calculate the mean RMSD for each portion, verifying whether the intervals are statistically comparable. We obtain the following results, 3.74±0.13Å, 4.05±0.17Å and 3.87±0.14Å respectively for the three parts, indicating a satisfactory condition of equilibrium. **(b)** Principal Component Analysis of the 500 MKK7 frames employing protein alpha carbon atoms. The structures extracted from the simulation quite homogeneously sample the space composed of the first two eigenvectors and no very well-defined clusters are present.

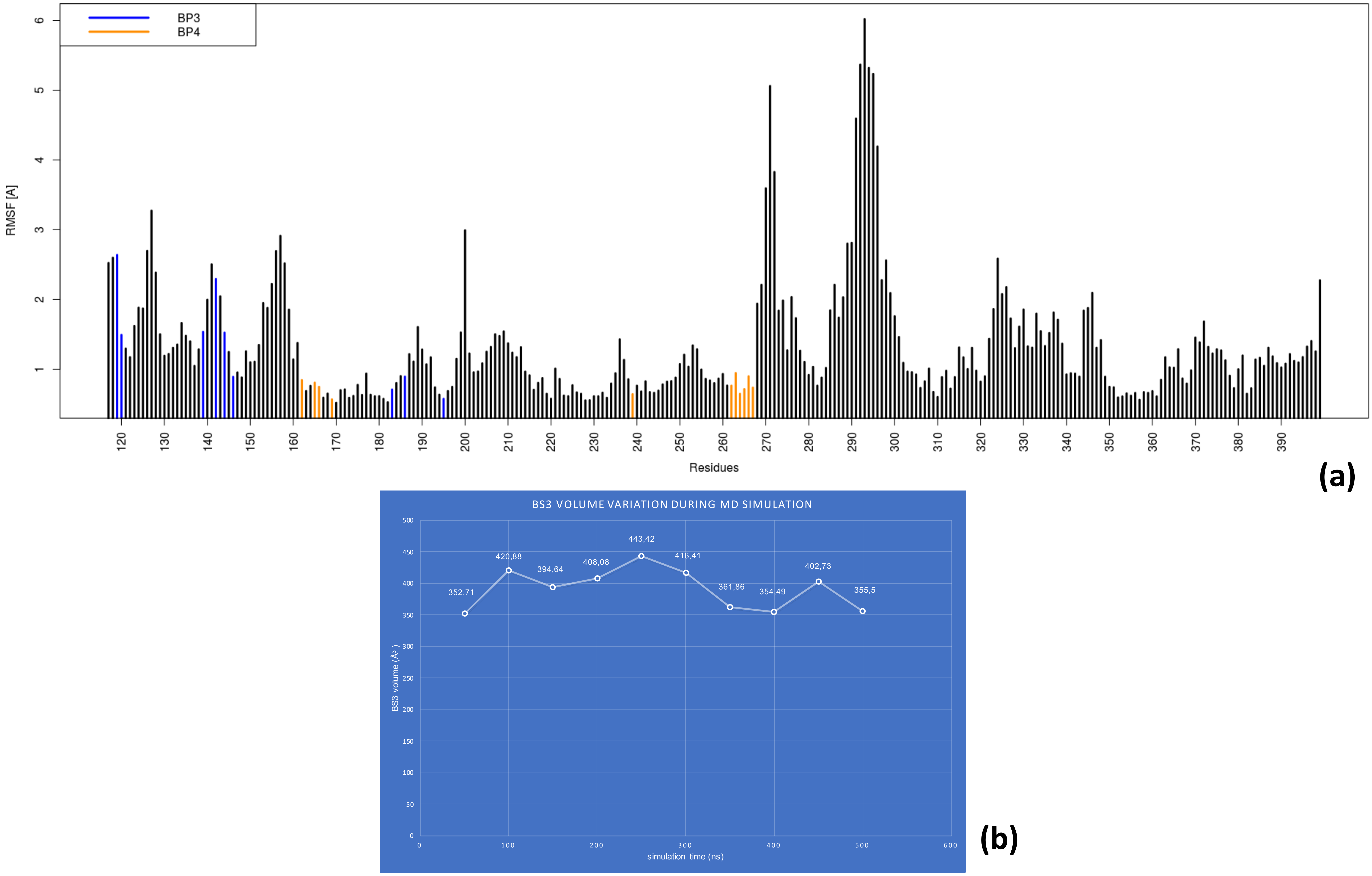

Figure S7: (a) Analysis of local conformational mobility of the BP3 (involving residues 119, 120, 139, 142, 144, 146, 183, 186, 195 and depicted in blue) and BP4 (involving residues 162,165, 166, 169, 239, 262-267 and depicted in orange). Root Mean Square Fluctuation (RMSF) values are reported. They capture, for each residue, the fluctuation about its average position. **(b)** BP3 cavity volume evolution during the simulation. BP3 volumes from 50ns to 500ns, one every 50ns, are reported. The average volume value is 385±12,7 Å^3.

Residue	NH	αCH	βCH	γCH	others
**tyr1**	-	4.30	2.77, 2.72		2,6H: 6.93 3,5H: 6.66
**arg2**	-	4.06	1.48, 1.40	1.23	δCH: 2.95
**phe3**	-	4.40	3.03, 2.83		2,4,6 H: 7.14 3,5 H: 7.23

Table S1. Chemical shifts (ppm) of DTP3 at peptide/KD R100 molar ratio. Peptide is N-terminal acetylated (Ac 1.83 ppm) and C-terminal amidated.

residue	NH	αCH	βCH	γCH	others
**arg1**	-	4.00	1.41	1.25	δCH: 2.94
**phe2**	-	4.44	2.86, 2.79		2,6 H: 7.06 3,5 H: 7.20 4H: 7.15
**tyr3**	-	4.30	2.88, 2.66		2,6H: 6.98 3,5H: 6.69

Table S2. Chemical shifts (ppm) of SCRB at peptide/KD R100 molar ratio peptide is N-terminal acetylated (Ac 1.85 ppm) and C-terminal amidated
